# Regulation of Neuronal APL-1 Expression by Cholesterol Starvation

**DOI:** 10.1371/journal.pone.0032038

**Published:** 2012-02-21

**Authors:** Mary Wiese, Adam Antebi, Hui Zheng

**Affiliations:** 1 Huffington Center on Aging and Department of Molecular and Human Genetics, Baylor College of Medicine, Houston, Texas, United States of America; 2 Max Planck Institute for Biology of Ageing, Köln, Germany; National Institutes of Health, United States of America

## Abstract

**Background:**

Alzheimer's disease (AD) is a neurodegenerative disorder characterized by the deposition of β-amyloid plaques composed primarily of the amyloid-β peptide, a cleavage product of amyloid precursor protein (APP). While mutations in APP lead to the development of Familial Alzheimer's Disease (FAD), sporadic AD has only one clear genetic modifier: the ε4 allele of the apolipoprotein E (ApoE) gene. Cholesterol starvation in *Caenorhabditis elegans* leads to molting and arrest phenotypes similar to loss-of-function mutants of the APP ortholog, *apl-1* (amyloid precursor-like protein 1), and *lrp-1* (lipoprotein receptor-related protein 1), suggesting a potential interaction between *apl-1* and cholesterol metabolism.

**Methodology/Principal Findings:**

Previously, we found that RNAi knock-down of *apl-1* leads to aldicarb hypersensitivity, indicating a defect in synaptic function. Here we find the same defect is recapitulated during *lrp-1* knock-down and by cholesterol starvation. A cholesterol-free diet or loss of *lrp-1* directly affects APL-1 levels as both lead to loss of APL-1::GFP fluorescence in neurons. However, loss of cholesterol does not affect global transcription or protein levels as seen by qPCR and Western blot.

**Conclusions:**

Our results show that cholesterol and *lrp-1* are involved in the regulation of synaptic transmission, similar to *apl-1*. Both are able to modulate APL-1 protein levels in neurons, however cholesterol changes do not affect global *apl-1* transcription or APL-1 protein indicating the changes are specific to neurons. Thus, regulation of synaptic transmission and molting by LRP-1 and cholesterol may be mediated by their ability to control APL-1 neuronal protein expression.

## Introduction

Alzheimer's disease (AD), the most common cause of dementia, is a progressive neurodegenerative disease defined by the deposition of amyloid plaques in the brain. These plaques are generated from a processing product of the amyloid precursor protein (APP), amyloid-β. While mutations in APP have been identified in familial AD cases, only one gene has been definitively linked to sporadic AD cases. The ε4 isoform of ApoE, a lipoprotein involved cholesterol trafficking, leads to increased susceptibility to AD and earlier onset of the disease [1], [2]. These observations have led to a large body of evidence supporting the modulation of Aβ production through changes in cholesterol homeostasis. However, it is still unclear whether these changes directly cause the increased susceptibility to AD seen in the individuals carrying the ε4 allele, or whether other pathways of cholesterol homeostasis are involved.

In the current study, we utilized the model organism *Caenorhabditis elegans* to determine the consequences of modulating sterol conditions and their effects on the *C. elegans* APP ortholog, *apl-1*. In *C. elegans*, sterols are necessary for two major developmental processes: dauer formation and molting [3], [4]. *C. elegans* lack the enzymes necessary to manufacture sterols and therefore must obtain them from their diet [5]. Worms deprived of either cholesterol or the low-density lipoprotein (LDL) receptor-related protein (LRP-1), a homolog of megalin thought to be involved in the endocytosis of sterols, develop a molting defect [3], [6]. Involvement of this lipoprotein receptor may hint at a molecular link with APP as members of the ApoE-binding, LDL receptor family, including LRP1, megalin (LRP2) or apolipoprotein E receptor 2 (ApoER2) have been shown to interact with APP and regulate its endocytic trafficking [7]. Since knock-down of *apl-1* expression leads to a similar molting phenotype as cholesterol deprivation and *lrp-1* knock-down, it is possible that APL-1 either has a role in cholesterol metabolism or is regulated by cholesterol [8], [9].

We report here that cholesterol starvation in *C. elegans* leads to hypersensitivity to the acetylcholine esterase inhibitor, aldicarb, revealing a defect in synaptic transmission. This defect is similar to that seen during *lrp-1* and *apl-1* knock-down. Also, loss of cholesterol leads to a decrease in APL-1 protein in the neurons suggesting that cholesterol is necessary for proper APL-1 protein expression. This regulation appears to be at the protein level as global *apl-1* transcription appears unaffected, as well as being specific to neurons. Together, these results suggest a role for cholesterol in the regulation of APL-1 protein expression in the neurons, potentially altering the ability of APL-1 to perform its function in molting or synaptic transmission.

## Results

### Loss of Dietary Cholesterol or LRP-1 Leads to Similar Molting and Neurotransmission Defects As *apl-1* Loss-of-Function

**Figure 1:**
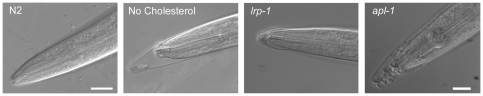
Similar molting defects are seen during cholesterol starvation and *apl-1* loss. N2 worm at the L2 stage compared to arrested L2 worms from the second generation grown on a cholesterol-free diet, *lrp-1(ku156)* mutant and *apl-1(tm385)* arrested at the L1/L2 molt. (Scale bars, 10 µm).

Cholesterol is a required component for the molting process in the worm and must be obtained through dietary intake [5], [10]. Cholesterol starvation leads to defects in the molting process manifesting in the worm's inability to shed its cuticle [3]. Worms with loss of *lrp-1* or *apl-1* through null mutation or RNAi have also been previously described as developing molting defects [3], [8], [9], [11]. The molting defects seen in mutants *lrp-1(ku156)*, *apl-1(tm385)* and worms experiencing cholesterol starvation are very similar to one another in that the worms exhibit loose cuticle around the head and tail, common with defects in the final molting stage of ecdysis (Figure 1) [12]. These similarities imply a functional interaction between APL-1 and cholesterol metabolism.

**Figure 2:**
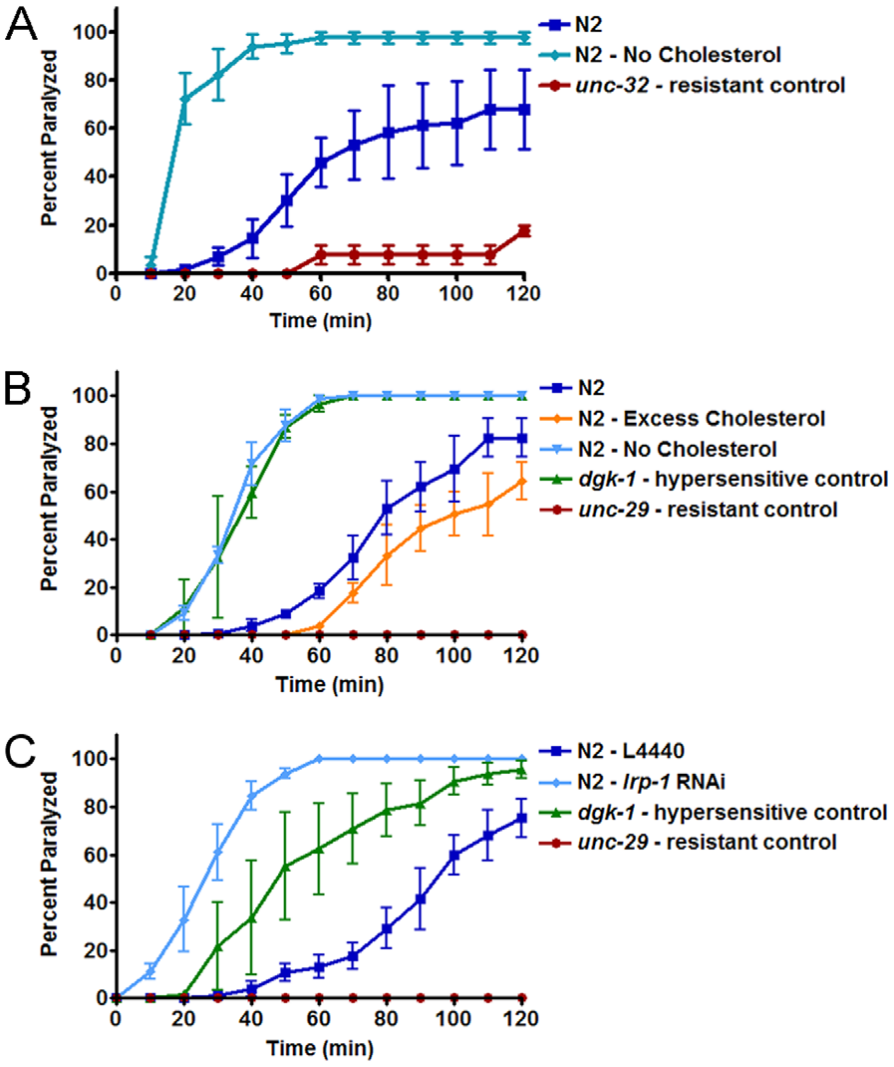
Aldicarb hypersensitivity in worms during cholesterol starvation or *lrp-1* RNAi. A) The second generation of worms on a cholesterol starvation diet arrest at the L1/L2 stage and are aldicarb hypersensitive. B) Worms grown on a cholesterol-free diet for one generation were tested on aldicarb as young adults. Excess cholesterol (50 µg/ml) had no effect on aldicarb sensitivity, however cholesterol starved animals are aldicarb hypersensitive. C) Worms grown on *lrp-1* RNAi also experience aldicarb hypersensitivity. Control strains for these experiments are as follows: *unc-32(e189)* - mutant for a vacuolar H^+^-ATPase, aldicarb resistant; *dgk-1(nu62)* - diacylglycerol kinase mutant, aldicarb hypersensitive; *unc-29(e1072)* - non-alpha subunit of the nicotinic acetylcholine receptor, aldicarb resistant. Each experiment was performed at least three times (n = 50 per strain). Error bars represent the s.e.m.

To test whether cholesterol starvation leads to defects in neurotransmission similar to that of *apl-1* loss [9], worms were grown on stringent cholesterol-free media and the second generation of progeny was tested for synaptic defects using the acetylcholine esterase inhibitor, aldicarb. Aldicarb prevents acetylcholine breakdown in the synaptic cleft of motor neurons, leading to paralysis over time [13], [14]. Defects promoting excess or depleted acetylcholine at the motor neuron synapse can be detected through hypersensitivity or resistance to aldicarb, respectively. Worms on the cholesterol starvation diet arrested at the L2 stage (Figure 1), therefore, controls were age-matched to the arrested L2 worms in this experiment. Extreme hypersensitivity to aldicarb was seen in arrested worms deprived of dietary cholesterol (Figure 2A). Young adult worms from the first generation of plates lacking cholesterol were also aldicarb hypersensitive (Figure 2B). Since these F1 worms did not have an apparent molting defect, this would suggest that the aldicarb sensitivity is not due to the molting defect per se, but rather is specific to a neurotransmission defect. We then tested young adult worms after developmental *lrp-1* knock-down and found the same aldicarb hypersensitivity (Figure 2C). The similarity of aldicarb hypersensitivity during cholesterol starvation, *lrp-1* RNAi and *apl-1* RNAi strengthens support for a regulatory link between cholesterol and *apl-1*.

We next tested whether excess cholesterol could rescue the aldicarb hypersensitivity seen during *apl-1* knock-down. Patients defective in intracellular cholesterol trafficking due to a mutation in the Neimann Pick type C gene, NPC1, experience progressive neurodegeneration and accumulation of cholesterol in lysosomal compartments [15]. The double mutant of the redundant *C. elegans* homologs to NPC1, *ncr-1* and *ncr-2*, is dauer constitutive, meaning these genes normally negatively regulate dauer formation [16]. The enhancement of dauer formation can be suppressed in this mutant by adding excess cholesterol to the media, bypassing the need for cholesterol redistribution within the cell [17]. However, we found that excess cholesterol cannot rescue the aldicarb hypersensitive phenotype seen during *apl-1* knock-down (Figure S1), suggesting that the aldicarb sensitivity is not due to deficiency in intracellular cholesterol transport.

### Lack of Cholesterol Promotes Loss of APL-1 Protein Expression in the Neurons

**Figure 3:**
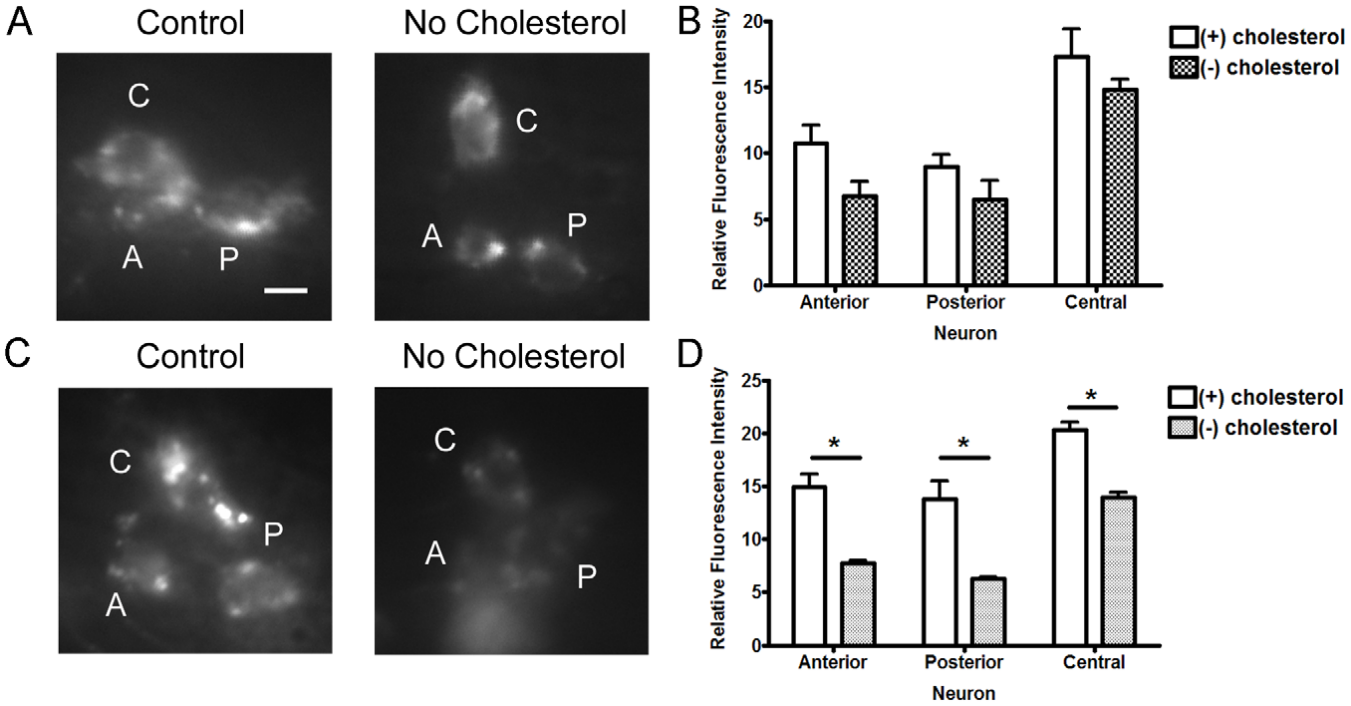
Lack of cholesterol leads to loss of APL-1::GFP in head neurons. A) Representative pictures of APL-1::GFP expression in three head neurons of L4 worms grown for one generation on plates with (+) or without (−) cholesterol. Head neurons are identified by A (anterior), P (posterior) or C (central). B) Quantification of APL-1::GFP fluorescence (n = 15 per experiment). C) Representative pictures of APL-1::GFP expression in L4 worms of the second generation grown on normal or cholesterol-deficient plates. D) Quantification of the neuronal cell body expression of APL-1 (n = 10–15 per experiment). Experiments were performed twice. (*, P<0.05) Error bars represent the s.e.m.

**Figure 4:**
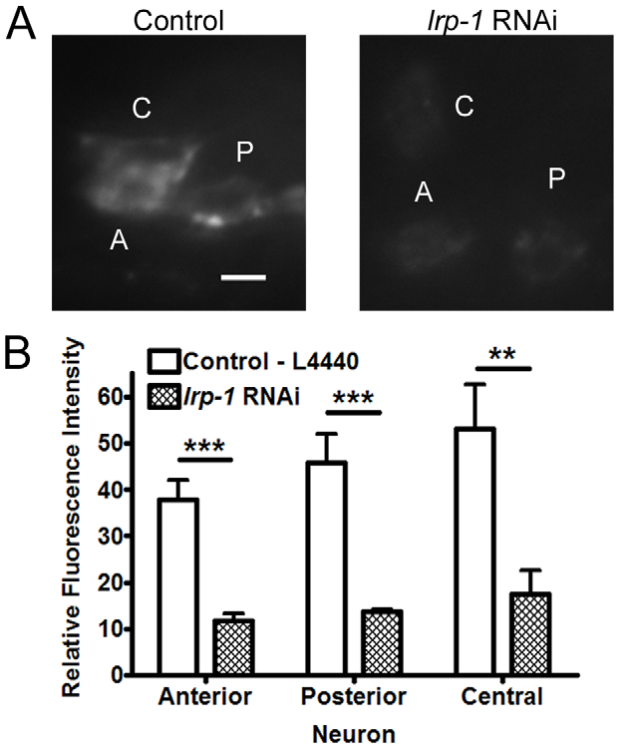
Knock-down of *lrp-1* leads to decreased APL-1 in head neurons. A) Representative pictures of the RNAi sensitive strain *nre-1 lin-15b* expressing *apl-1::gfp* on either control or *lrp-1* RNAi at the L4 stage. B) Fluorescence quantification of APL-1::GFP in the neuronal cell body. (n = 10–12) Experiments were performed twice. (**, P<0.01, ***, P<0.001) Error bars represent the s.e.m.

To determine if cholesterol deprivation affects APL-1, we examined worms expressing APL-1::GFP cultivated on NGM with and without cholesterol. To avoid the molting defect, we used less stringent cholesterol conditions, which do not lead to arrest in the second generation. The first generation of worms grown in these conditions began to show a slight, but non-significant, decrease in APL-1::GFP expression in the head neurons where *apl-1* is normally expressed (Figure 3A, B). By the second generation on cholesterol-deficient media, a clear decrease in APL-1::GFP was seen (Figure 3C, D). When testing APL-1::GFP expression in worms developed on *lrp-1* RNAi, we saw the same drop in APL-1 expression (Figure 4). However, the same strain placed on media containing excess cholesterol showed no difference in APL-1::GFP expression in the same head neurons (Figure S2).

### A Cholesterol Poor Diet Leads To Minimal Changes of Global APL-1 Transcription and Protein Levels

**Figure 5:**
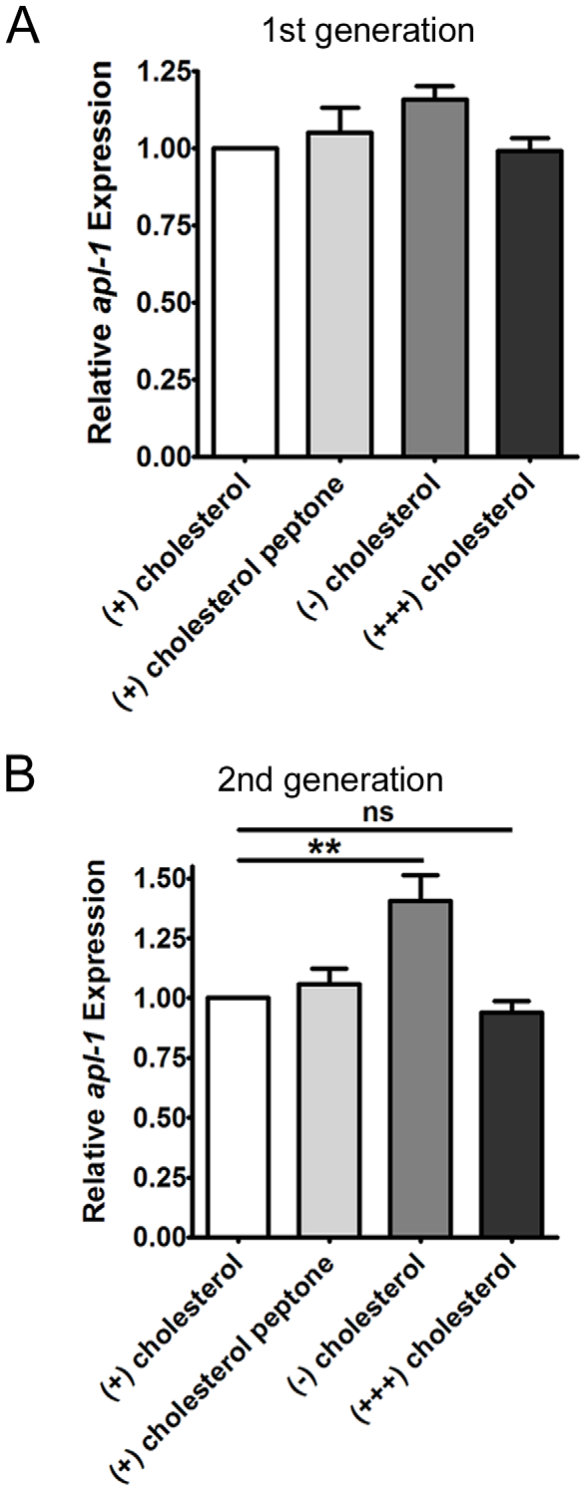
Cholesterol starvation has little effect on *apl-1* transcription. A) *apl-1* expression levels of N2 L4 worms grown on cholesterol-deficient or excess cholesterol conditions for one generation. Cholesterol peptone indicates cholesterol containing control plates that, like the (−) cholesterol plates, were made using ether extracted peptone. The slight increase in *apl-1* expression was not significant this generation. B) Expression of *apl-1* in the second generation of worms grown on the different cholesterol conditions. (n = 3 experimental replicates) (**, P<0.01) Error bars represent the STD.

Depriving worms of cholesterol leads to a decrease in APL-1::GFP, therefore we were interested in determining whether this was due to a decrease in *apl-1* transcription. Depriving worms of dietary cholesterol led to slight increases, rather than a decrease, of *apl-1* transcription in both the first and second generations (Figure 5). Excess cholesterol had no effect on *apl-1* transcription (Figure 5). Since we were detecting transcription in a worm population, it is possible that there are changes in specific tissues that we were unable to detect. However, overall *apl-1* transcription does not appear to be regulated by cholesterol.

Western blots revealed that on both cholesterol-deficient media and the more stringent cholesterol starvation, the overall amount of APL-1 protein remains constant (Figure S3). This suggests that APL-1 loss under these conditions is specific to the neurons, thereby contributing to the neurotransmission defect.

## Discussion

Cholesterol and cholesterol trafficking proteins have been associated with changes in APP processing as well as AD pathology [1], [2]. To further explore the relationship between APP and cholesterol, we investigated the phenotypic similarities between cholesterol starvation and loss of *lrp-1* to *apl-1* loss-of-function in *C. elegans*. The similarity in molting phenotypes between worms experiencing cholesterol starvation, *lrp-1* knock-down and *apl-1* knock-down supports the hypothesis that the three are functionally linked. We found that they also all possessed strikingly similar neurotransmission defects, however the role of cholesterol in synaptic transmission in the worm is unclear. Using the intrinsically fluorescent dehydroergosterol (DHE), Matyash et al. detected cholesterol in certain neurons in the nerve ring as well as the pharynx, spermatheca, excretory gland cells, proximal oocytes and embryos [18]. However, DHE was not found in other sets of neurons in the worm. Since cholesterol is detected on only a few specific membranes in the worm, coupled with the fact that worms need only a small amount of cholesterol to survive, the likelihood that the cholesterol requirement for survival can be attributed to its role in membrane dynamics is thought to be low [19]. Rather, the major function of cholesterol in the worm is predicted to come from its ability to be manufactured into sterol hormones as worms have at least 284 predicted nuclear hormone receptors [20], [21], [22]. One such hormone is the bile acid like steroid, dafachronic acid, which works through the nuclear receptor DAF-12 [23]. However, other steroid dependent receptors have yet to be identified. Therefore we were interested in determining whether APL-1 may be upstream of cholesterol regulation or downstream through regulation by steroid hormones.

We found that cholesterol deprivation lowers APL-1::GFP expression in the head neurons and that the same loss of APL-1::GFP is seen in worms on *lrp-1* RNAi. During *apl-1* knock-down, the worms develop synaptic defects [9], suggesting that loss of APL-1 in these neurons under either cholesterol starvation or *lrp-1* RNAi may contribute to the synaptic defect observed in both these conditions.

Intracellular cholesterol transport mutant phenotypes can be rescued by exposure to excess cholesterol, however, cholesterol cannot rescue the *apl-1* knock-down synaptic defect or molting defect, suggesting that *apl-1* is not involved in intracellular transport of cholesterol. Nor is it likely that APL-1 acts as a major cholesterol receptor to bring extracellular cholesterol to specific cell types. Since the soluble N-terminus of APL-1 can rescue the molting defects and lethality of the *apl-1* mutant [8], it seems unlikely that the major function of APL-1 would be to transport cholesterol into the cell as a cell bound receptor.

Cholesterol and *apl-1* have potentially been linked through the ability of the nuclear hormone receptor NHR-25 to regulate *apl-1* transcription in the seam cells, possibly through the action of a sterol derived ligand [24]. However, we found minimal changes in total *apl-1* transcription during cholesterol deprivation. This indicates that either NHR-25 does not have a steroid based hormone, as is true with its mammalian homologs SF-1 and LHR-1 [25], [26], or NHR-25 does not regulate transcription of *apl-1* specifically in the neurons. Indeed, NHR-25 expression appears to be confined to the hypodermis, seam cells and the developing gonad [24], [27]. We also saw a small increase in global *apl-1* transcription on stringent cholesterol starvation conditions over two generations (data not shown) that may be due to compensation effects from the loss of APL-1 protein in the neurons. While transcriptional regulation of *apl-1* within the neurons remains unclear, gross *apl-1* transcription does not appear to rely on the presence of cholesterol and therefore, sterol hormones.

In the same way, total protein in the worm does not appear to be affected, opening the interesting possibility that the loss of APL-1 protein in the neurons is tissue specific. Since neuron-specific expression of *apl-1* has been reported to rescue the lethality and molting defect of an *apl-1* null mutant [8], it is possible that loss of APL-1 within neurons during cholesterol starvation may be a large contributor to the subsequent phenotypes of molting, arrest and synaptic transmission defects. Cholesterol may be regulating the endocytosis or processing of APL-1, thereby affecting its ability to properly regulate molting and synaptic transmission.

In summary, we demonstrated the phenotypic similarities between cholesterol starvation and loss-of-function of both *lrp-1* and *apl-1*, each of which have similar molting and neurotransmission defects. Worms on a cholesterol-deficient diet experienced a loss of APL-1 protein in head neurons, however this loss is not likely due to transcriptional regulation. Total APL-1 protein in the worms also remained consistent over varying cholesterol conditions, indicating that cholesterol may regulate APL-1 protein specifically in the head neurons, but not other tissues. The ability of LRP-1 and cholesterol to regulate synaptic transmission and molting may be mediated, at least in part, by their ability to control APL-1 neuronal protein expression.

## Methods

### *C. elegans* Strains

Strains were cultivated at 20°C as described previously [13]. The wild-type Bristol N2 strain, *lrp-1(ku156)*, *apl-1(tm385)/lon-2(e678)*, *unc-32(e189)*, *dgk-1(nu62)*, *unc-29(e1072)*, *nre-1(hd20) lin-15b(hd126)* and AA1504 (dhEx544[*papl-1::apl-1::gfp*]) were used in this study.

### Cholesterol and Aldicarb Experimentation

NGM plates were made containing 5 µg/ml cholesterol (normal) or no cholesterol (cholesterol-deficient). The peptone in both control and cholesterol-deficient conditions was ether extracted as described to remove any residual sterols [10]. The plates were seeded with the OP50 bacterial strain that was washed 5× to remove any trace sterols present in the growth media. For cholesterol starvation conditions, cholesterol-free plates were made as previously described [4]. In brief, peptone and cholesterol were excluded from the NGM plates and agarose replaced agar. These plates were seeded as normal, with the exception that the OP50 cultures were washed 5× in M9 and then concentrated 20 fold due to the decreased growth of the bacteria on peptone-free media. Excess cholesterol plates were made using NGM with 50 µg/ml cholesterol. To test neurotransmission defects, worms either in their first or second generation, young adult and L2 stages respectively, on regular or cholesterol starvation conditions were tested on seeded plates containing their corresponding cholesterol treatment and 1 mM aldicarb (PS734; Sigma-Aldrich). Worms were tested for paralysis every 10 minutes for 2 hours using a harsh touch. Animals were scored as paralyzed if unable to respond to the touch.

### RNAi

RNAi experiments were performed using RNAi clones isolated from the Ahringer RNAi library (Gene Service) as described [28]. The *lrp-1* RNAi clone was a kind gift from the Rogaev lab. Clones were streaked onto 10 µg/ml tetracycline and 100 µg/ml ampicillin, then individual colonies were selected for overnight cultures in LB containing 100 µg/ml ampicillin. The cultures were used to seed NGM plates containing 1 mM IPTG and 50 µg/ml ampicillin.

### Fluorescence Microscopy and Quantification

Imaging was performed by placing live animals anesthetized with 30 mg/ml BDM (2,3-Butanedione monoxime; Sigma-Aldrich) on a 2% agarose pad as previously described [29]. Images were obtained using a Zeiss Axioscope 2plus upright microscope equipped with an Axiocam MRm camera and Axiovision 4.1 software. Pictures were acquired with a 100× or 63× lens. Head neuron fluorescence was quantified by imaging neurons of 10–20 worms per genotype with identical exposure times. The neuronal cell bodies were imaged at their widest diameter in the plane of focus. Control pictures were taken on the same day to account for differences in bulb intensity. Fluorescence intensity of the neuronal cell bodies was quantified using ImageJ software (National Institutes of Health) and then cholesterol or RNAi conditions compared using the Student's t-test.

### RNA Extraction and qRT-PCR

RNA was collected and qRT-PCR performed as previously described [9]. In brief, worm populations were synchronized by bleaching and then harvested at the L4 stage. After four freeze/thaw cycles, RNA was isolated using the Qiagen RNeasy Lipid Tissue kit method with the addition of the RNase free DNase steps (Qiagen). cDNA was generated using Superscript III First Strand kit (Invitrogen). PCR primer sequences to test *apl-1* expression are as follows: apl-1 Fwd ACTCACAGTGTCAGACCGTACCA and apl-1 Rev GTGCGGGACTTGAAGAGCTT. *ama-1* expression was used as the endogenous control. The Power SYBR Green PCR Master Mix protocol (Applied Biosystems) was used to perform quantitative real-time PCR (qRT-PCR) with the ABIprism 7000 and data collected using the 7000 System SDS Software (Applied Biosystems). Primer efficiencies were validated using the standard curve method, then all subsequent experiments were performed with a cDNA dilution of 1∶50 using the comparative C_t_ method for analysis. Bars represent the mean of three experiments, each containing a different biological sample with error bars calculated from the sample and endogenous control standard deviations (STD = √((Sample STD)∧2+(Housekeeping Gene STD)∧2)). Gene expression under different cholesterol conditions were compared statistically using one-way ANOVA with Bonferroni post-hoc test.

### Protein Extraction and Western Blotting

Worms were synchronized by bleaching and samples harvested as previously described [9], [30] In brief, L4 stage worms were washed with TE, pelleted, then placed at −80°C for 15 min. RIPA buffer containing protease inhibitors (Roche) was added, then the samples were sonicated and centrifuged at 10,500× g for 10 min at 4°C. Supernatants were obtained and protein concentrations were measured by plate reader using a detergent-compatible colorimetric protein assay (Bio-Rad). After 2× loading buffer was added, samples were boiled at 80°C for 10 min prior to loading. SDS-PAGE was performed using 5% SDS-polyacrylamide gel loaded with 20 µg of protein per sample. Nitrocellulose membrane was used for transfer and then blocked for 2 hrs in 5% milk diluted with PBST. The membrane was probed with anti-GFP antibody 1∶5000 (ab290; Abcam) to detect the APL-1::GFP fusion protein and *C. elegans* γ-tubulin antibody 1∶1000 (ab50721;Abcam) as a loading control. Membranes were washed with PBST and incubated with 2° anti-Rabbit HRP-conjugated antibody 1∶5000 (Vector Laboratories). After additional washes in PBST, bands were detected using the Amersham ECL chemiluminescence reagent (GE Healthcare Life Sciences). Band density was quantified using ImageJ software. Band densities were normalized to the loading control and compared using the Student's t-test.

## Supporting Information

Figure S1 **Synaptic defects seen during** ***apl-1*** **knock-down cannot be rescued by excess cholesterol.** RNAi sensitive strain *rrf-3(pk1426)* was grown on plates containing *apl-1* RNAi and excess cholesterol conditions and then tested on aldicarb. Each experiment was performed three times. (n = 50 per strain) Error bars represent the s.e.m.
(TIF)

Figure S2 **Excess cholesterol has no effect on APL-1::GFP expression.** A) Fluorescence levels within the head neurons of APL-1::GFP expressing worms in the first generation grown on excess cholesterol (n = 15 per experiment) B) Fluorescence levels of APL-1::GFP in the second generation of worms grown on excess cholesterol (n = 15 per experiment). No significant differences were seen after applying the Student's t-test analysis. Each experiment was performed three times. Error bars represent the s.e.m.
(TIF)

Figure S3 **Global APL-1 protein is unchanged after cholesterol is eliminated from the diet.** A) Representative Western blot of synchronized worm populations in their first and second generations on a cholesterol-deficient diet. B) Quantification of Western samples showing the relative protein is unchanged. C) Protein levels were also unchanged in the first and second generations on a cholesterol-free diet. D) Quantification of Western blot. (n = 3) Error bars represent the s.e.m.
(TIF)
