# Supplementary figures and images for: Regulation of Neuronal APL-1 Expression by Cholesterol Starvation
Source: PLoS One. 2012 Feb 21;7(2):e32038. doi: 10.1371/journal.pone.0032038 (PMC3283687; doi:10.1371/journal.pone.0032038)

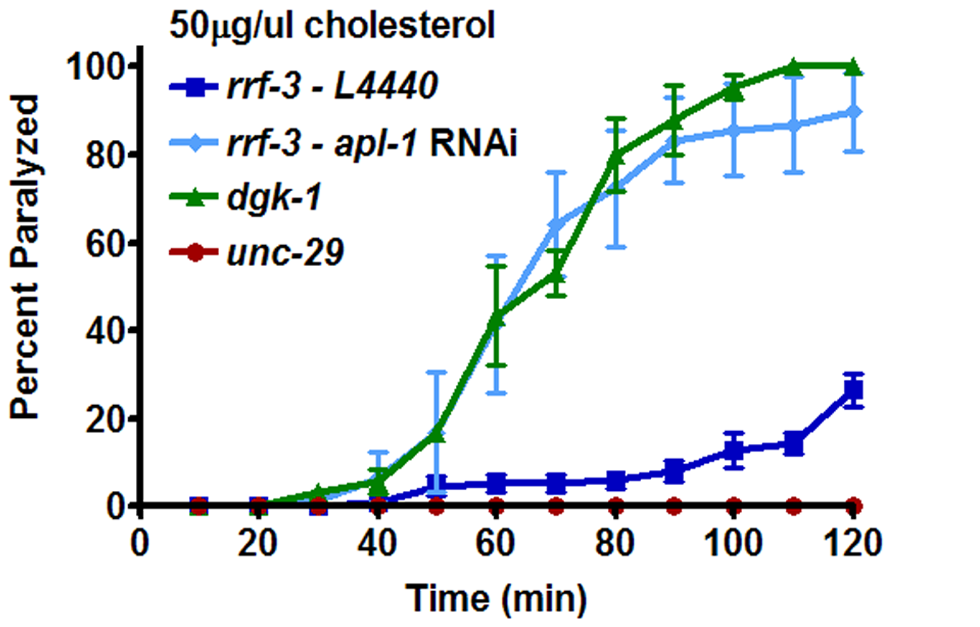

Supplement: Figure S1 — Synaptic defects seen during apl-1 knock-down cannot be rescued by excess cholesterol. RNAi sensitive strain rrf-3(pk1426) was grown on plates containing apl-1 RNAi and excess cholesterol conditions and then tested on aldicarb. Each experiment was performed three times. (n = 50 per strain) Error bars represent the s.e.m. (TIF) [file pone.0032038.s001.tif]

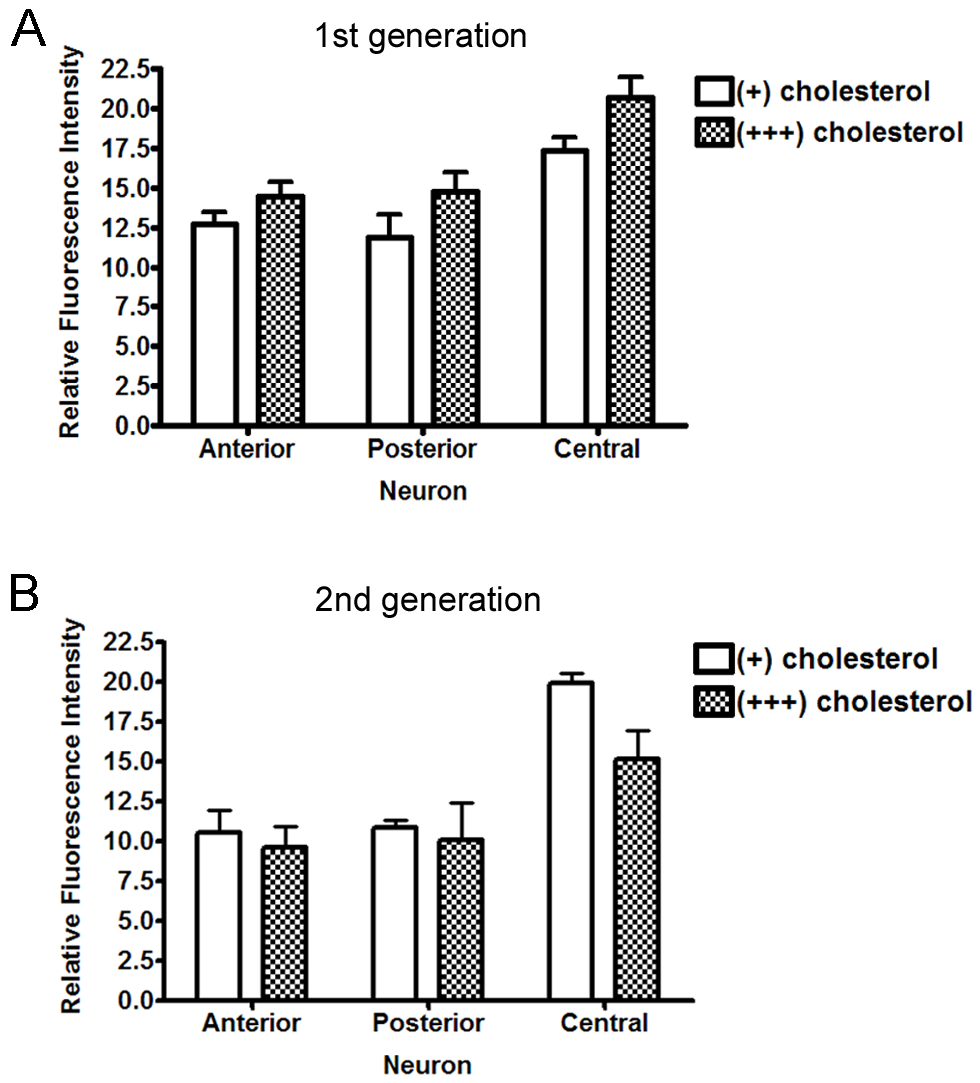

Supplement: Figure S2 — Excess cholesterol has no effect on APL-1::GFP expression. A) Fluorescence levels within the head neurons of APL-1::GFP expressing worms in the first generation grown on excess cholesterol (n = 15 per experiment) B) Fluorescence levels of APL-1::GFP in the second generation of worms grown on excess cholesterol (n = 15 per experiment). No significant differences were seen after applying the Student's t-test analysis. Each experiment was performed three times. Error bars represent the s.e.m. (TIF) [file pone.0032038.s002.tif]

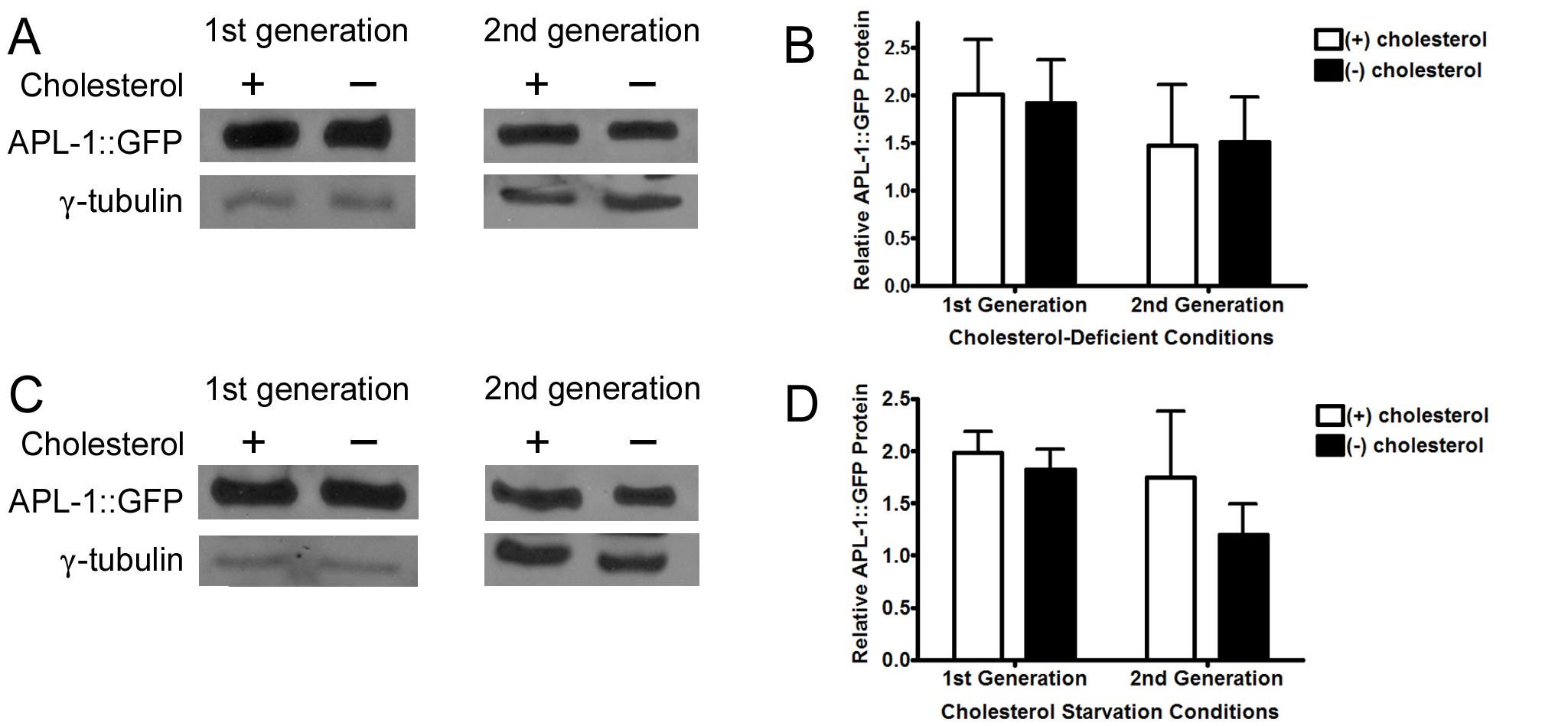

Supplement: Figure S3 — Global APL-1 protein is unchanged after cholesterol is eliminated from the diet. A) Representative Western blot of synchronized worm populations in their first and second generations on a cholesterol-deficient diet. B) Quantification of Western samples showing the relative protein is unchanged. C) Protein levels were also unchanged in the first and second generations on a cholesterol-free diet. D) Quantification of Western blot. (n = 3) Error bars represent the s.e.m. (TIF) [file pone.0032038.s003.tif]
